# Isoform-specific effects of ApoE on neurite outgrowth in Olfactory Epithelium culture

**DOI:** 10.1186/1423-0127-20-49

**Published:** 2013-07-12

**Authors:** Aseem Hussain, Minh Luong, Apryl Pooley, Britto P Nathan

**Affiliations:** 1 Department of Biological Sciences, Eastern Illinois University, 600 Lincoln Avenue, Charleston, IL 61920, USA

**Keywords:** ApoE, Neurite outgrowth, Neuronal differentiation, Olfaction, Olfactory neurons, Low-density lipoprotein receptor-related protein, Receptor-associated protein, Alzheimer’s disease

## Abstract

**Background:**

The apolipoprotein E4 (apoE4) genotype is a major risk factor for developing late-onset Alzheimer’s disease (AD). Inheritance of apoE4 is also associated with impairments in olfactory function in early stages of AD. In this project we examined the effects of the three common isoforms of human apoE (apoE2, apoE3, and apoE4) on neuronal differentiation and neurite outgrowth in explant cultures of mouse olfactory epithelium (OE).

**Results:**

The OE cultures derived from apoE-deficient/knockout (KO) mice have significantly fewer neurons with shorter neurite outgrowth than cultures from wild-type (WT) mice. Treatment of the apoE KO culture with either purified human apoE2 or with human apoE3 significantly increased neurite outgrowth. In contrast, treatment with apoE4 did not have an effect on neurite outgrowth. The differential effects of human apoE isoforms on neurite outgrowth were abolished by blocking the low-density lipoprotein receptor-related protein (LRP) with lactoferrin and receptor-associated protein (RAP).

**Conclusion:**

ApoE2 and apoE3 stimulate neurite outgrowth in OE cultures by interacting with the lipoprotein receptor, LRP. ApoE4, the isoform associated with AD, failed to promote neurite outgrowth, suggesting a potential mechanism whereby apoE4 may lead to olfactory dysfunction in AD patients.

## Background

Apolipoprotein E (apoE) is a protein component of several lipoproteins [1]. ApoE functions in the redistribution of lipids by binding to the low-density lipoprotein (LDL) receptor and the LDL receptor-related protein (LRP) family members [2]. Receptor-lipoprotein binding initiates internalization and degradation of lipoproteins, making lipids available for use in the regulation of intracellular lipid metabolism. ApoE ranges in length from 279 to 310 residues, with a high degree of sequence similarities among species [3]. There are three major isoforms of apoE in humans differing by amino acids at positions 112 and 158 [4]. The most common isoform, apoE3, contains cysteine and arginine at positions 112 and 158, respectively [3]. Both positions have cysteine in apoE2 and arginine in apoE4. Mice have one form of apoE, which is similar to human apoE3 in its structural and functional properties, including receptor binding and lipoprotein preferences [3].

Numerous genetic studies have revealed that inheritance of apoE4 allele increases the risk and rate of progression of late-onset Alzheimer disease [5-9]. About 65-80% of AD patients have at least one apoE4 allele [9,10]. In addition, apoE4 inheritance decreases the age of onset of AD [8,9]. Neurofibrillary tangles and amyloid plaques, the two hallmarks of AD, are increased in brain samples from apoE4 carriers as compared to non-apoE4 carriers [11,12]. Both plaques and tangles appear earlier in apoE4 patients as compared to non-apoE4 patients. In addition, AD patients with apoE4 genotype showed decreased dendritic growth, reduced synaptic numbers, and widespread degeneration of neurons in areas of the brain related to learning and memory, as compared to non-apoE4 patients [13]. Numerous hypotheses have been proposed to explain apoE4 effects on AD; however, the mechanism whereby apoE4 leads to AD is still unclear.

Inheritance of the apoE4 genotype is also associated with olfactory dysfunction including deficits in odor fluency, odor identification, odor recognition and olfactory threshold sensitivity [14-18]. These olfactory impairments are observed early in the course of AD, even before the onset of clinical dementia [14]. Non-demented apoE4-individuals showed significant decline in olfactory processing as compared to individuals without apoE4 allele [19,20]. The AD patients with apoE4 allele showed greater deficits in olfactory tests than siblings without apoE4 allele [21]. Data from longitudinal studies also have shown that apoE4 inheritance is associated with poor scores in olfactory tests [15]. In essence, these findings show that apoE plays a key role in olfactory function.

The mechanism underlying apoE effects on olfactory function is not clear. In previous studies, we showed that apoE is expressed at high levels by a variety of cell types in the olfactory epithelium and its underlying lamina propria [22]. ApoE was localized in the basal cell layer, suggesting that it could promote neurogenesis by facilitating differentiation of basal cells to mature neurons. In addition olfactory nerve regeneration following olfactory nerve lesioning was significantly slower in apoE KO mice as compared to WT mice, suggesting that apoE may play a critical role in olfactory nerve regeneration in mice [23].

In this project we explored this possibility by examining the effects of apoE isoforms on neuronal differentiation and neurite outgrowth in olfactory epithelium (OE) explant cultures. We found that (1) OE cultures derived from apoE KO mice have significantly fewer neurons with shorter neurite outgrowth than cultures from WT mice; (2) treatment of apoE KO cultures with either purified apoE2 or apoE3 significantly increased neurite outgrowth, whereas treatment with apoE4 had no effect; and (3) the differential effects of human apoE isoforms on neurite outgrowth were abolished by blocking the low-density lipoprotein receptor-related protein (LRP) with lactoferrin and receptor associated protein (RAP).

## Methods

### Olfactory explant epithelial culture

Homozygous apoE KO mice (C57BL/6-Apoe^<tmiUnc>^) bred 10 generations onto C57BL/6 background and control mice (C57BL/6 J) were obtained from Jackson Laboratory (Bar Harbor, MA). Cell culture medium, including Neurobasal A, Hanks’ Balanced Salt Solution, B-27 Supplement and FGF2 were obtained from Invitrogen Corporation (Grand Island, NY). Glutamine and fibronectin were purchased from Sigma Chemicals (St. Louis, MO). Costar Brand Tissue Culture 24-well plates were purchased from Fisher Scientific (Chicago, IL).

Prior to each experiment, glass slips were coated with 50 μg/ml fibronectin solution (Invitrogen Grand Island, NY) for two hours at 37°C. For each experiment, seven to eight post-natal pups (2 days old) were decapitated using a sterile surgical scissors and their nasal cavity was cut open sagitally, exposing the OE. The OE was carefully dissected and placed in ice-cold 10 ml of Hanks’ Balanced Salt Solution (Invitrogen Grand Island, NY), containing gentamycin (100 μg/ml) and glucose (6 mg/ml). The OE was sliced using sterile razor blade into approximately 200 μm thick explants. The explants were transferred into Neurobasal-A (NBA) media (Invitrogen Grand Island, NY) containing B27 supplement (20 μl/ml) and glutamine (0.5 mM). The explants were transferred to a 24-well plate containing the fibronectin coated slips, and the explants were incubated for 30 minutes without any media in a humidified incubator at 37°C and 5% CO_2_. Following incubation, 500 μl of growth media (Neurobasal-A medium with 5 ng/ml FGF_2_ and B27) was added to each well and the plate was further incubated. New growth media was changed every two days. Cultures were fixed at 8 days *in vitro* (DIV).

### Measurement of neuronal numbers, halo size, and neurite outgrowth

The OE cultures from WT and apoE KO mice were grown for 8 days in growth media, fixed with 4% paraformaldehyde, and cultures were immunostained for tubulin III (neuronal marker) as described below. The number of neurons, radii of the inner and outer halos, and combined length of the short and the long neurite outgrowth was measured using a stage micrometer mounted on an Olympus BX50 fluorescent microscope. A minimum of 60 neurons was measured for each treatment condition. To avoid bias in measurements, all neurons in the visual fields located at 5 quadrants (center, northeast, northwest, southeast and southwest) of the culture on cover slips was measured. In addition, the researcher was unaware of the genotype (WT versus apoE KO) and/or the treatment condition.

In experiments with apoE, recombinant human apoE were purchased from Panvera (Madison, WI), and dialyzed overnight in 0.1 M ammonium bicarbonate. The OE cultures at 2 DIV were incubated with medium alone or with apoE isoforms (5 μg/ml). The media was replaced every two days with re-addition of apoE. At 8 DIV, the cultures are fixed, immunostained for tubulin III, and neurite outgrowth measured as described above.

For studies with LRP inhibitors, lactoferrin was obtained from Sigma Chemical (St. Louis, MO), and purified receptor associated protein (RAP) was generously provided by Dr. Dudley Strickland (American Red Cross, Rockville, MD). The OE cultures at 2 DIV were pre-incubated for 1 h with medium alone or with either RAP (5 μg/ml) or lactoferrin (10 μg/ml). Following incubation, apoE isoforms (5 μg/ml) were added to the medium. The media was replaced every two days with re-addition of the test reagents. At 8 DIV, the cultures are fixed, immunostained for tubulin III, and neurite outgrowth measured as described above.

### Immunocytochemistry

Immunostaining of neuronal marker, tubulin III, was performed as previously described [24,25]. Briefly, cover slips of cultures at 8 DIV were rinsed with warm phosphate buffered saline (PBS) and fixed with 4% paraformaldehyde in PBS for 15 min at room temperature. Cells were permeabilized with 0.5% Triton in PBS for 10 minutes. Cells were rinsed with PBS and blocked with 5% donkey serum and 0.05% Triton in PBS for 60 minutes. Cells were incubated with mouse anti-tubulin III (Sigma Chemicals, St. Louis, MO) at 1:200 dilution in blocking solution for 2h at room temperature. Following incubation, cells were rinsed three times with PBS and incubated with TRITC-conjugated donkey anti-mouse (Jackson ImmunoResearch West Grove, PA) in blocking solution at 1:200 for 1h. Cells were rinsed with PBS and were mounted with mounting medium (Vector Laboratories, Burlingame, CA). Immunoreactive cells were counted and photographed on an Olympus BX50 microscope with appropriate excitation filters.

Immunocytochemistry of olfactory sensory neurons using markers for mature (OMP) and immature (GAP43) was performed as described above for tubulin III. At 8 DIV cells were fixed, permeabilized, and blocked with 1% BSA for 10 minutes. Cells were incubated overnight at 4°C with goat anti-OMP (Wako labs, Wako TX) at 1:500 dilution in 4% donkey serum or with rabbit anti-GAP43 (Millipore, Billerica, MA) at 1:200 dilution in 4% rabbit serum. Following incubation, cells were rinsed with PBS and incubated for 1h at room temperature with Cy3-conjugated donkey anti-goat at 1:500 dilution in 4% donkey serum or with FITC-conjugated donkey anti-rabbit at 1:1000 dilution in 4% rabbit serum. Cells were washed, mounted on glass slides, and photographed as described above for tubulin III. All controls with no primary were negative.

### Statistical analysis

All experiments were repeated at least four times using different preparations of OE cultures and reagents. The data in individual experiments were presented as mean ± standard error, and statistical analysis (One way ANOVA, Post-hoc Bonferroni Corrected t-tests) was performed using Sigmaplot software.

## Results and discussion

### Characterization of olfactory epithelium cultures

**Figure 1:**
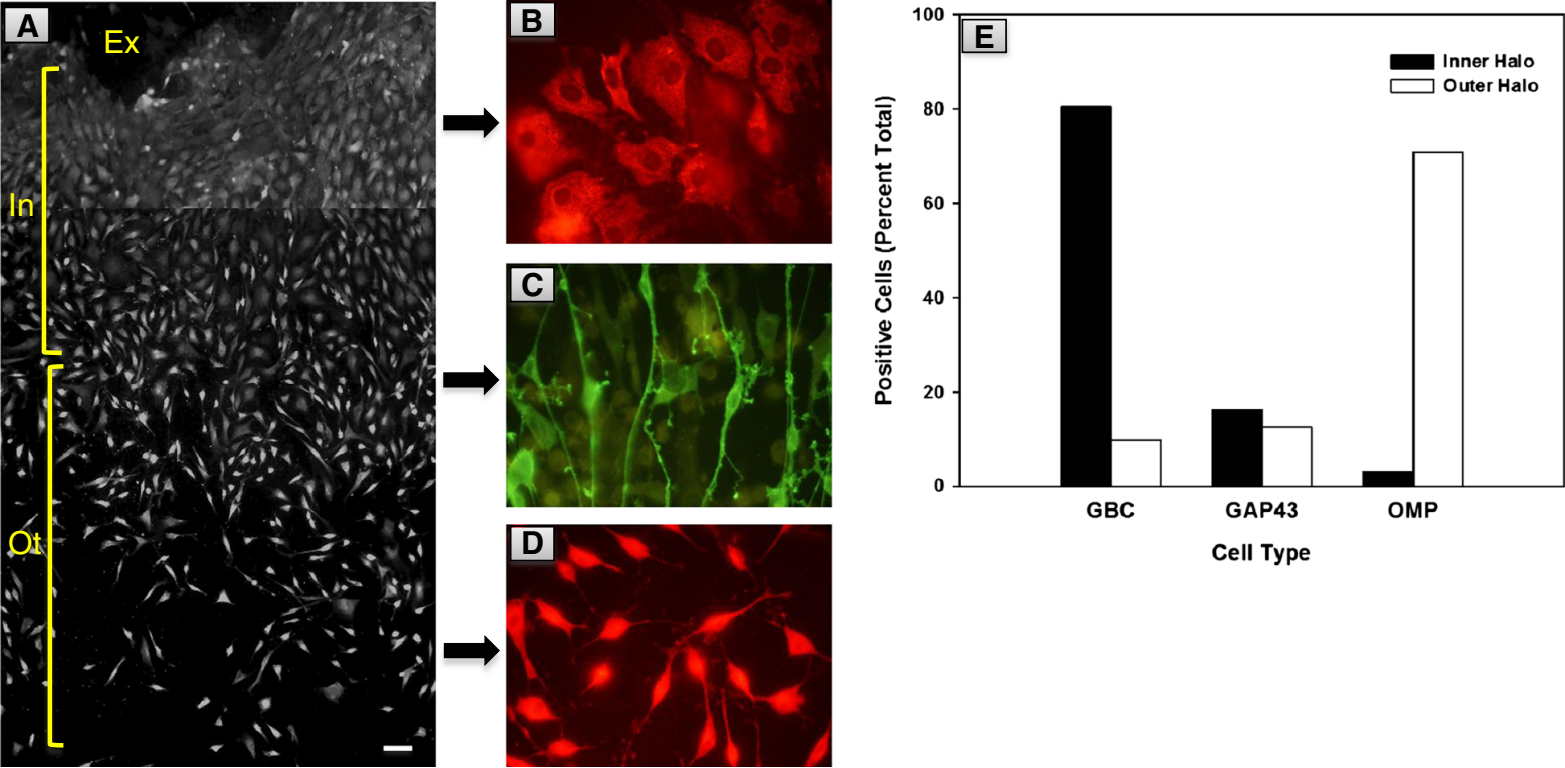
**Characterization of the OE culture. (A)** Two contiguous phase contrast pictures of the OE culture at 8 DIV were merged to depict the location of the explant (Ex), and the inner (In) and outer (Ot) halos. Scale bar = 50 μm. Representative morphologies of cells immunostained for GBC1 **(B)**, GAP43 **(C)** and OMP **(D)** in the OE culture. **(E)** Quantification of the cell types in the inner and outer halos in OE cultures.

We used a modified protocol to culture olfactory epithelium (OE) cells derived from post-natal mice [26,27]. At 4 days *in vitro* (DIV) the neuronal precursors and sensory neurons migrate out of the explant and establish as two large halos (Figure 1). The inner halo, which is closer to the explant, is primarily composed of densely populated, irregular shaped cells. About 80% of the cells in the inner halo were positive for GBC-1, a marker for globose basal cells in the mature OE [28,29]. Most of the cells in the boundary of the inner and the outer halo stained for the growth-associated protein (GAP) 43, a marker for immature olfactory sensory neurons in the OE [30-32]. The cells in the outer halo were bipolar in shape and were sparsely distributed. These cells expressed olfactory marker protein (OMP), a marker for mature sensory neurons in the OE [33,34]. In essence, the OE culture technique described herein provides an *in vitro* model system to study the various cell types that normally resides in the OE.

### ApoE promotes differentiation of basal cells to olfactory sensory neurons

**Figure 2:**
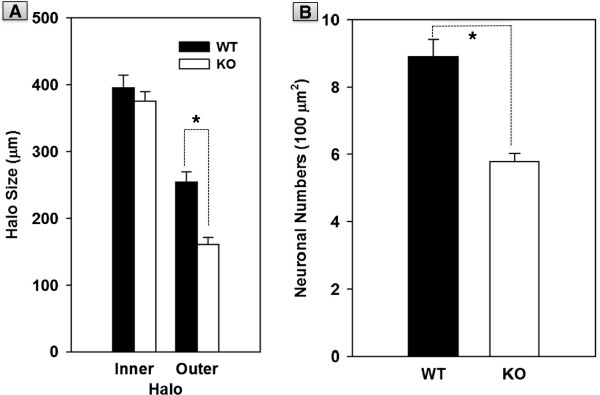
**Effects of apoE deficiency on halo size and neuronal numbers in OE cultures. (A)** Inner halo size was comparable in OE cultures derived from WT and apoE KO mice. In contrast, outer halo was significantly (* p < 0.001) larger in WT mice cultures than in apoE KO cultures. **(B)** Neuronal numbers were also significantly (* p < 0.001) higher in the WT cultures versus apoE KO cultures.

We first compared halo size in OE cultures from apoE KO mice with that from age- and strain-matched WT mice (Figure 2A). The size of the inner halo, which is primarily composed of GBC-1^+^ basal cells, was comparable in WT and apoE KO cultures. In contrast, the size of the outer halo, which is primarily composed of cells with bipolar outgrowths, was significantly (p < 0.001) smaller in the apoE KO mice than that in the WT mice cultures. To directly test if apoE deficiency leads to decreased neuronal numbers, we performed tubulin III immunocytochemistry, which is a marker for neurons [35-37]. Fewer tubulin III positive cells were in the outer halo of the KO mice than that in the WT mice culture (Figure 2B). These data suggest that apoE deficiency in the apoE KO mice leads to reduced differentiation of basal cells to sensory neurons in the OE cultures.

Our results are consistent with previous studies that have also shown a critical role for apoE in neuronal differentiation. ApoE is known to modulate factors that are important for neurogenesis, including WNT2 and granulin [38,39]. In addition, apoE also promotes survival of neurons in normal and injured nervous system by up regulating pro-neurogenic factors like Bcl2 [40]. The precise mechanism whereby apoE promotes basal cell differentiation to olfactory sensory neurons is not clear, and has to be examined in future studies.

### ApoE facilitates neurite outgrowth in OE cultures

**Figure 3:**
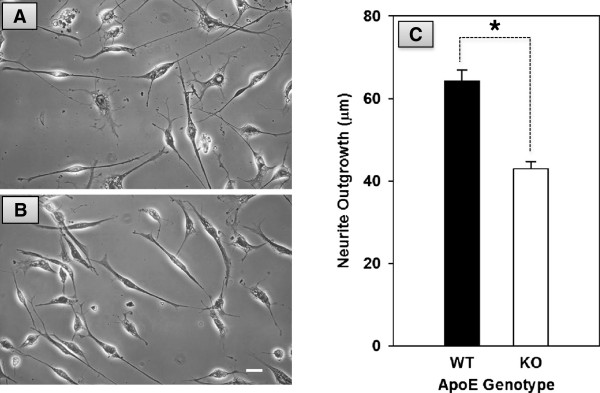
**Effects of apoE deficiency on neurite outgrowth in OE cultures.** Phase contrast photographs of representative neurons in OE cultures derived from WT **(A)** and apoE KO mice **(B)**. Cultures were grown for 8 days, fixed and photographed. Scale bar = 25 μm. **(C)** Quantification of the effects of apoE deficiency on neurite outgrowth. Neurite outgrowth was significantly (* p < 0.001) longer in WT cultures than in apoE KO cultures.

To examine if apoE is important for neuronal process outgrowth we measured neurite outgrowth at 8 DIV. Our measurements revealed that neurons in the apoE KO cultures had significantly (p < 0.001) shorter neurite outgrowth than those from neurons in the WT cultures (Figure 3). These results are consistent with previous studies that showed diminished neurite outgrowth in embryonic and adult neuronal cultures derived from apoE KO mice [25,41]. ApoE may have increased neurite outgrowth directly by redistributing lipids released from degenerating olfactory explant to newly differentiated neurons that are in dire need for lipids to extend neurites. Alternatively, apoE could have indirectly modulated neurite outgrowth by modulating factors that are critical for extension of neuronal processes.

### Human apoE isoforms have differential effects on neurite outgrowth in apoE KO cultures

**Figure 4:**
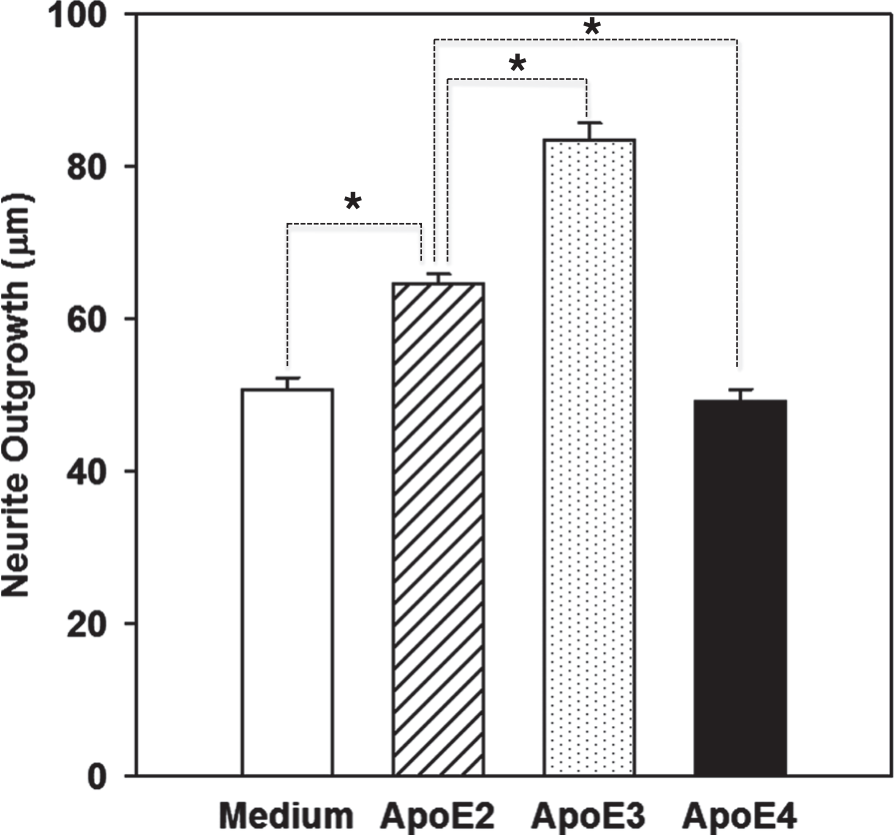
**Effects of human apoE on neurite outgrowth in OE cultures.** Two-days old cultures from apoE KO mice were grown in medium alone, and in medium containing 5 μg/ml of human apoE2, human apoE3, or human apoE4. Medium was changed every two days, and apoE re-added. Cultures were fixed at 8 DIV, and neurite outgrowth (* p < 0.05) was measured in tubulin III immunopositive neurons as described under Methods.

We next examined the effects of purified human apoE on neurite outgrowth in OE cultures derived from apoE KO mice. Purified human apoE (5 μg/ml) was added to a 2 DIV culture. The medium was replaced every two days and apoE was re-added. Neurite outgrowth was measured at 8 DIV. Cultures incubated with apoE2 had significantly longer neurite outgrowth as compared to cultures grown in medium alone (Figure 4). Similarly, apoE3 treated cultures had significantly longer neurite outgrowth than those cultures treated with apoE2 or medium alone. In contrast, apoE4 treatment did not have an effect, with neurite outgrowth comparable to those in cultures incubated with medium alone. This finding is in striking contrast with other studies that have shown that apoE4 decreases neurite outgrowth in cell lines, and dissociated cell culture systems [25,42-44]. The reasons for this discrepant result are not clear, but differences in culture paradigm, that is, explant versus dissociated neuronal cultures, and culture medium composition could have contributed to this anomaly.

### The LRP mediates the isoform-specific effects of apoE on neurite outgrowth in OE cultures

**Figure 5:**
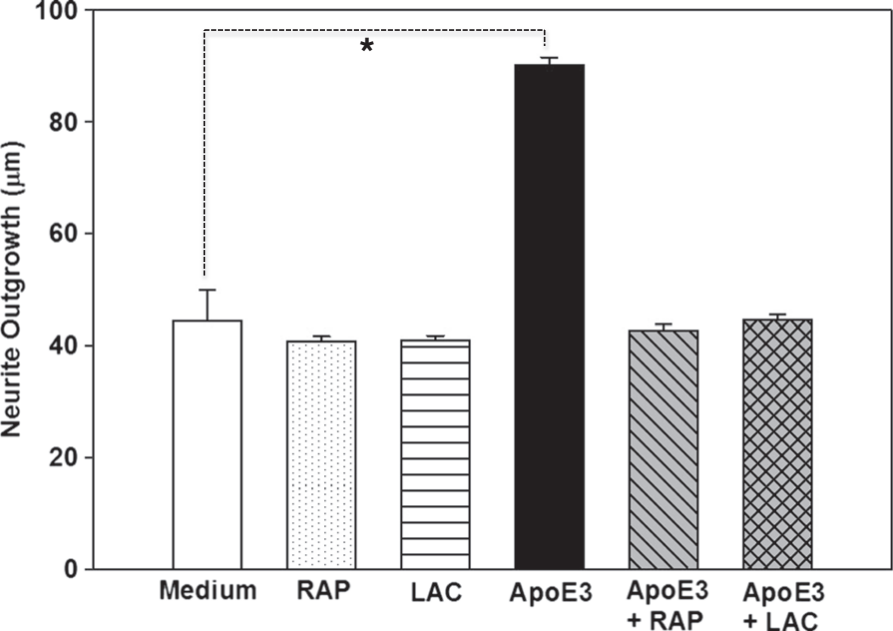
**Effects of blocking lipoprotein receptor, LRP, on differential effects of human apoE on neurite outgrowth in OE cultures.** Two-days old cultures from apoE KO mice were incubated for 1 h in medium alone or in medium containing receptor associated protein (RAP) (5 μg/ml) or lactoferrin (LAC, 10 μg/ml). Following incubation with RAP or LAC, human apoE isoform (5 μg/ml) was added to the medium and incubation was continued for a total of 8 days. Medium was changed every two days, and test reagents re-added. Cultures were fixed, and neurite outgrowth (* p < 0.05) was measured in tubulin III immunopositive neurons as described under Methods.

Previous studies have shown that LRP, a major lipoprotein receptor, plays a critical role on neuronal structure and function, including neuronal differentiation and process outgrowth [25,45-47]. Therefore, we examined if the effects of apoE on neurite outgrowth is mediated by the LRP. In this experiments we blocked the LRP using lactoferrin and RAP, and then examined if human apoE3 treatment can increase neurite outgrowth in OE cultures [48-50]. Lactoferrin and RAP, at the concentration used in this study, did not have an effect on neurite outgrowth (Figure 5). However, blocking of the LRP with RAP or lactoferrin abolished the neurite outgrowth promoting effect of apoE3, and the length of neurites in apoE3 treated cultures were similar to cultures grown in medium alone. These data suggest that the effects of apoE3 on neurite outgrowth are mediated through the LRP pathway of lipoprotein uptake.

How apoE isoforms differentially modulate neurite outgrowth by using the LRP is unclear. One possibility is that apoE isoforms that are internalized through the LRP are differentially processed in neurons. For example, we previously reported that apoE3 accumulated in both the cell bodies and neurites, whereas, apoE4 accumulated to a lesser extent only in the cell body [44,51,52]. The differential accumulation and localization of apoE isoforms resulted in isoform-specific effects on neuronal microtubules that are critical for process growth. Fewer well-formed microtubules, and a greatly reduced ratio of polymerized to monomeric tubulin were observed in apoE4-treated neurons than did neurons treated with apoE3 [44]. Whether or not the effect of apoE isoforms on neurite outgrowth is due to their differential regulation of neuronal cytoskeleton in the OE culture has to be examined in future studies.

## Conclusion

The results from this study showed that apoE plays a critical role in differentiation and neurite outgrowth in olfactory sensory neurons. Moreover, human apoE-isoforms differentially modulated neurite outgrowth. The apoE2 and apoE3 stimulated neurite outgrowth in OE cultures by interacting with the lipoprotein receptor, LRP. In contrast, apoE4, the isoform of apoE that is associated with AD, failed to facilitate neurite outgrowth.

Previous studies have shown that apoE4 individuals have a significant decline in odor threshold and odor identification, and have delays in processing of olfactory information [14-17,53]. The mechanism underlying these isoform-specific effects of apoE on olfactory function is not clear, but based on results from this study it is tempting to suggest that the inability of apoE4 to foster neurite outgrowth may, in part, underlie olfactory dysfunction in AD. Together, these data suggest a tremendous role for apoE in neurological health, which is modulated by apoE genotype.

## Abbreviations

ApoE: Apolipoprotein E; AD: Alzheimer’s disease; WT: Wild-type; LRP: Low-density lipoprotein receptor related protein; KO: Knockout; RAP: Receptor associated protein; LAC: Lactoferrin.

## Competing interests

The authors declare that they have no competing interests.

## Authors’ contributions

AH, ML, and AP carried out the research work, prepared the data for publication, and drafted the manuscript. BPN conceived of the study, and participated in its design and coordination and helped to draft the manuscript. All authors have read and approved the final manuscript.
